# Importance of frontal sinus radiographs for human identification

**DOI:** 10.1016/S1808-8694(15)31396-3

**Published:** 2015-10-17

**Authors:** Rhonan Ferreira da Silva, Rodrigo Naves Pinto, Geovane Miranda Ferreira, Eduardo Daruge Júnior

**Affiliations:** 1 MSc in Forensic Dentistry at FOP/UNICAMP, Forensic Investigator at Polícia Técnico-Científica, GO; 2 Specialist in Vascular Surgery, Forensic Physician at Polícia Técnico-Científica, GO; 3 DDS, Resident of Maxillofacial Surgery and Trauma at DOD/UEM; 4 PhD in Sciences, Professor of Forensic Dentistry at FOP/UNICAMP. Instituto Médico-Legal da Polícia Técnico-Científica do Estado de Goiás

**Keywords:** forensic anthropology, frontal sinus, radiography

## INTRODUCTION

Humans beings can be identified through a series of methods, being fingerprints the most widely adopted when soft tissue is preserved. However, when the cadaver is carbonized or in skeletal form, forensic dental and anthropologic analysis may become necessary to identify the individual^1^. Medical documentation (mainly x-ray images) may substantially improve the chances of identifying corpses initially deemed unrecognizable. This paper describes a forensic case in which human remains could be positively correlated to the identity of a missing person through analysis of images of the subject’s frontal sinus in skull posterior-anterior x-ray images.

## CASE PRESENTATION

Parts of a human skeleton were found in a patch of woodlands in December of 2006. Many of the remaining bones - ulna, ribs, vertebrae etc - were fractured. The skull had sings of trauma and carbonization. Only the cranial vault was relatively preserved, while face bones were fractured and detached from their joints. Preliminary anthropologic analysis revealed the corpse had characteristic traits of an adult woman.

Police investigation indicated that the tentative victim was a Caucasian woman of 30 years of age missing since May of 2006. She had a head injury when she was 8, and was clinically followed up until she was 25.

**Figure 1:**
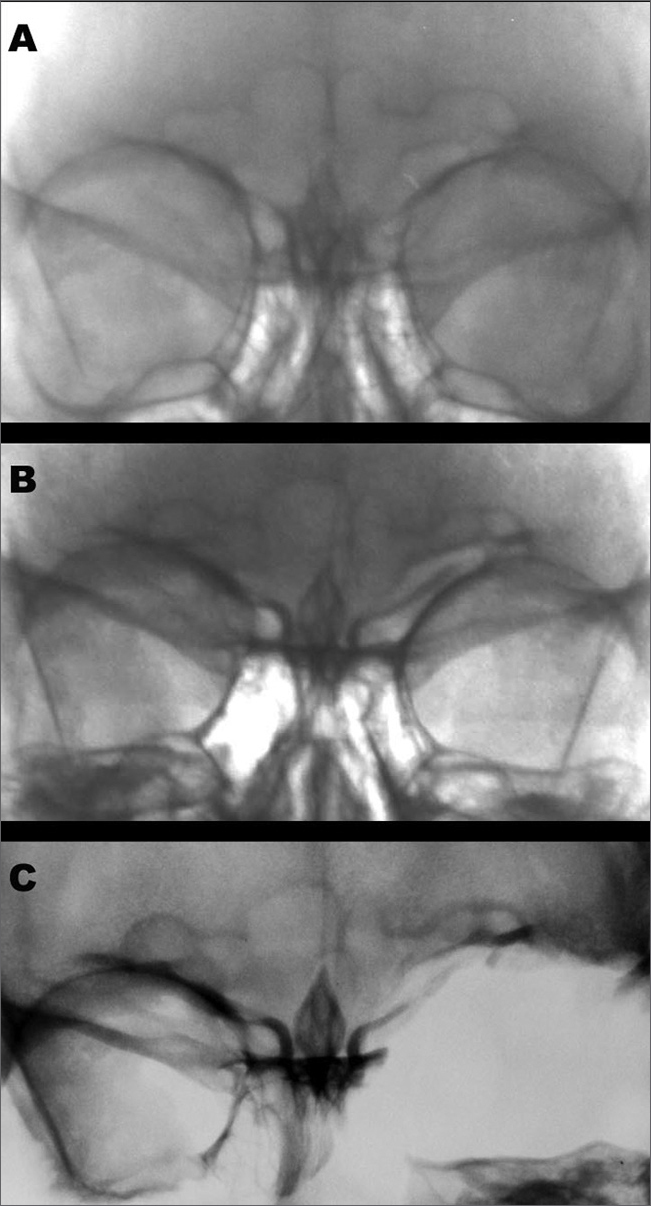
Images of the victim’s frontal sinus in 1989(A), 1993(B), and post-mortem in 2006(C).

The forensics team was given a series of images to try and determine whether the remains belonged to the missing person. Among them were two skull posteroanterior x-ray images from 1989 and 1993 (Fig. 1A and 1B). X-ray images of the skull remains were then taken to support anthropologic comparison (Fig. 1C).

## DISCUSSION

The frontal sinuses are pneumatic cavities lined with mucosa situated between and internal and external laminae of the frontal bone^2^. They become radiologically evident at age 5–6, and are fully developed at age 10–12^3^.

The frontal sinus is absent in only 4% of the population and presents distinctive variations in shape, area, and symmetry, thus becoming an important parameter to determine gender dimorphism^4^ and allow subject identification^5^. Posteroanterior x-ray images taken under proper processing standards and obtained through adequate technique are essential for good forensic practices^6^.

## CLOSING REMARKS

The identity of the mortal remains was found to be that of a missing woman were identified as the x-ray images produced while she was alive were properly taken, thus enabling frontal sinus morphological analysis. The x-ray images were digitized and adjusted for brightness and contrast to allow improved contour visualization. The results from the comparison between the x-ray images taken before and after the subject’s death indicated the existence of converging traits in both sides of the face, although the left orbit was fractured (Fig. 1C). Aside from their use in the medical practice in identifying frontal sinus trauma and disease, posteroanterior x-ray images allow the visualization of the morphology of the area thus supporting forensic identification efforts.
